# Mfd interacts with RNA polymerase to modulate flagellum-dependent motility, chemotaxis, and population heterogeneity in the stationary phase of *Bacillus subtilis*

**DOI:** 10.1128/jb.00589-25

**Published:** 2026-05-14

**Authors:** Jessica Grifaldo, Hilda Leyva-Sanchez, Khristian Rosales, Amber Callaway, Andres Sotelo, Holly Anne Martin, Ryan King Perez, Jocelyn Chavez Rios, Elizabeth N. Huezo, Mario Pedraza-Reyes, Eduardo A. Robleto

**Affiliations:** 1 School of Life Sciences, University of Nevada 169168, Las Vegas, Nevada, USA; 2 Department of Physical and Life Sciences, Nevada State University 15633, Henderson, Nevada, USA; 3 Division of Natural and Exact Sciences, Department of Biology, University of Guanajuato 428638, Guanajuato, Mexico; University of Massachusetts Chan Medical School, Worcester, Massachusetts, USA

**Keywords:** *Bacillus subtilis*, swimming motility, transcription of coding sequences

## Abstract

*Bacillus subtilis* is a model for cell differentiation, capable of transitioning between distinct states: a sessile, chained state (often associated with biofilm formation), and a motile, planktonic state. This transition is governed by a complex regulatory network that includes alternative sigma factor-D (σ^D^), which drives the expression of genes for autolysins, flagellar biosynthesis, and chemotaxis, resulting in separated, flagellated, motile cells. Although the conserved transcription-coupled repair factor mutation frequency decline (Mfd) is best known for resolving stalled RNA polymerase (RNAP) during DNA repair, its involvement in cell differentiation during the stationary phase remains poorly understood. This research shows that in the absence of Mfd translocation or RNAP recruitment, RNAP completes transcription of motility genes more frequently, despite unchanged σ^D^ transcript levels. Increased transcription of the σ^D^-dependent motility regulon led to greater flagellation; however, swimming motility and pH taxis were paradoxically reduced, indicating that Mfd is required to translate flagellar gene expression into functional motility. Notably, Mfd-deficient cells failed to maintain chained subpopulations, indicating an additional role in sustaining population heterogeneity. These findings reveal a previously unrecognized function of Mfd as an RNAP-modulating factor that coordinates motility gene expression, thereby expanding our understanding of how transcription-coupled repair proteins influence bacterial physiology and behavior under non-proliferative conditions.

**IMPORTANCE:**

The mutation frequency decline (Mfd) enzyme mediates transcription-coupled repair in transcribed genes by directly interacting with RNA polymerase (RNAP) during transcription of coding sequences. However, whether the Mfd factor regulates gene expression associated with adaptations unrelated to DNA repair and mutagenesis remains vague. Here, we show that Mfd regulates the completion of full transcripts of genes that confer swimming motility, chemotaxis, and cell heterogeneity in *Bacillus subtilis*. Furthermore, we identified the Mfd’s translocase activity and its interaction with RNAP as key elements of this regulation. Therefore, Mfd’s importance in bacterial physiology and adaptation goes beyond DNA repair.

## INTRODUCTION

Transcription-coupled repair (TCR) is a process that preferentially repairs DNA lesions in the template strand of actively transcribed genes over lesions in the non-template strand or non-actively transcribed DNA regions. In its canonical role, Mfd, short for Mutation frequency decline, was discovered to be a TCR factor that links transcription to DNA repair (1). Mfd translocates along dsDNA via ATP hydrolysis (2). When Mfd encounters a stalled RNA polymerase (RNAP) at a bulky-DNA lesion site, it can remove RNAP from the DNA template. Using its UvrB homology, Mfd recruits UvrA (3) and begins nucleotide excision repair (NER).

In addition to its role in TCR, Mfd contributes to the cellular stress response. Mfd-deficient strains show increased sensitivity to oxidative agents, such as hydrogen peroxide and nitric oxide, indicating that Mfd protects DNA. Also, Mfd-deficient cells are sensitive to diamide, implicating this factor in response to protein oxidation stress (4, 5). Interestingly, the protection against diamide operates independently of both NER and base excision repair (BER) pathways. Mfd also plays a role in promoting mutagenesis under nutritional stress (6–8). It contributes to stationary-phase mutagenesis, particularly in cooperation with MutY and other repair factors, and has been shown to interact with GreA to facilitate transcriptional bypass of spontaneous DNA lesions during nutritional stress (7). These functions are thought to help generate genetic diversity during times of environmental challenges. Finally, Mfd has been implicated in developmental and adaptive processes, such as sporulation and spore outgrowth (9, 10). During spore germination, Mfd works with proteins, such as DisA and UvrA, to repair DNA damage and support genome stability. Moreover, Mfd contributes to the development of antibiotic resistance in some bacterial species by promoting mutagenesis pathways that lead to heritable resistance (11).

In addition, Mfd modulates transcription independent of DNA damage by rescuing paused or backtracked RNAP complexes (12, 13). This activity influences transcription elongation and termination, particularly during transcription that generates structured mRNA molecules (14). Mfd has also been shown to associate broadly with RNAP genome wide, suggesting a general role in regulating transcription dynamics under various conditions (15, 16). In our previous RNA-Seq analysis of *Bacillus subtilis* cells lacking Mfd (15), we observed altered expression of numerous genes, including upregulation of those within the *fla/che* operons, which confer flagellar biosynthesis and chemotaxis, prompting us to investigate a potential role of Mfd in the control of bacterial motility.

*B. subtilis* motility is a finely tuned process; flagellar biosynthesis is hierarchically organized at the genetic level, yet subject to multi-layered feedback that determines when and how flagella are produced and activated. Central to this network is the 27 kb *fla/che* operon, which encodes 32 genes required for assembly of the flagellar basal body as well as the alternative sigma factor (σ^D^) (17, 18). The expression of this operon is controlled by several factors, including DegU, SwrA, YmdB, and the SinR/SlrR system (19). Importantly, σ^D^ itself is encoded at the distal end of the operon and drives a positive feedback loop that enhances expression of the *fla/che* genes and activates σ^D^-dependent operons such as *motAB* and *hag* (19, 20). Activation by SwrA–DegU and repression by the SinR–SlrR complex create a bistable switch, ensuring that only a subpopulation of cells expresses motility genes at any given time. Nutrient availability further modulates motility through CodY, a global repressor that binds at three sites within the operon; it binds a promoter-proximal site and two intragenic “roadblock” sites, resulting in decreased initiation and elongation of transcription (21). Mutations at these sites relieve repression, elevate σ^D^ activity, and shift the bistable culture toward a highly motile single-cell phenotype (21). Collectively, these inputs illustrate how motility is shaped not only by hierarchical promoters and sigma-factor feedback but also by global transcriptional regulators that modulate RNAP dynamics across this exceptionally long operon.

Besides this regulatory hierarchy, transcription elongation factors play a direct role in maintaining motility gene expression. NusA and NusG assist RNAP processivity and prevent premature termination across the *fla/che* operon, and loss of either factor disproportionately reduces the expression of distal genes, such as *sigD* and *hag*, leading to defects in flagellation and swimming motility (22). On the other hand, our previous RNA-Seq analysis, showing that Mfd deficiency results in upregulation of transcription of the *fla/che* operon (15), suggests that Mfd controls the completion of transcripts of motility genes.

Although the role of Mfd in DNA repair and RNAP rescue is well studied, it remains unclear how Mfd specifically influences the transcription dynamics of motility genes and whether this is achieved through premature termination of these σ^D^-dependent genes or via another mechanism. Furthermore, it remains unclear how the structural domains of Mfd are responsible for their different physiological roles in cells. Using gene expression analyses, mRNA stability assays, site-directed mutagenesis, functional assays, and bioinformatics, this study investigated how Mfd regulates the transcription of motility and chemotaxis genes in *B. subtilis*, determined the contributions of specific Mfd functional domains (such as the RNAP interaction vs. translocase activity) in its newly recognized physiological roles, and explored whether sequence or structural features of DNA and mRNA underlie Mfd-mediated regulation. Overall, these studies revealed that Mfd plays a previously unrecognized role in regulating transcription beyond promoter activation of motility and chemotaxis genes in *B. subtilis*. By linking transcription dynamics to cellular behaviors, such as swimming and pH sensing, our work highlights Mfd as a key factor in how bacteria allocate resources and adapt to stress. These findings expand the physiological significance of Mfd beyond DNA repair and establish it as a central regulator of bacterial adaptation.

## MATERIALS AND METHODS

### Bacterial strains and growth conditions

The parental strain, YB955, is a prophage-cured *B. subtilis* strain 168 derivative (23) and is routinely isolated on Luria Broth plates. Liquid cultures were grown in Penassay broth (PAB) (antibiotic medium 3, Difco Laboratories, Sparks, MD, United States), grown at 37°C and 220 rpm with aeration. When required, tetracycline (Tet; 10 μg⋅mL^–1^), spectinomycin (Sp; 100 μg mL^–1^), ampicillin (Amp; 100 μg⋅mL^–1^), erythromycin (Em; 1 μg mL^–1^), kanamycin (Kan; 10 μg mL^–1^), or isopropyl-β-d-thiogalactopyranoside (IPTG; 0.3 mM) was added to the media.

### Construction of mutant strains and Mfd variants

To construct the *motA^−^* and *sigD^−^* strains, genomic DNA was isolated from the corresponding BKE (*Bacillus* Knockout Erythromycin collection [24]) strains using the Wizard Genomic DNA Purification Kit (Promega, Madison, WI, USA). Isolated genomic DNA was then transformed into YB955 using competence procedures for *Bacillus* described previously (25). Briefly, YB955 was grown to T_90_, 90 min after the cessation of growth (the stationary phase), in GM1 broth (0.5% dextrose, 0.1% yeast extract, 0.2% casein hydrolysate, essential amino acids 50 μg/mL, and 1× Spizizen salt solution), and then diluted 10-fold into GM2 broth (GM1 broth plus 50 μM CaCl_2_, and 250 μM MgCl_2_). After 1 h of incubation at 37°C with aeration, genomic DNA (100 ng) was added. Cells were plated on LB medium containing kanamycin (10 μg/mL) to select for the Bacillus Knockout Kanamycin (BKK) allele. *sigD^−^* and *motA^−^* mutants were screened for loss of motility by inoculating a single colony on plates with 0.3% agar (26).

To construct the double mutant strain (*cheV^−^ cheW^−^),* genomic DNA was first isolated from the BKK strain carrying the *cheV*^−^ mutation and transformed into the parental YB955. Cells were plated on LB medium containing kanamycin (10 μg/mL) to maintain the BKE allele. The transformants were verified by PCR using (cheV FW/cheV RV) primers, and the strain was named JG101. Genomic DNA from the BKE strain carrying the *cheW^−^* mutation was isolated and transformed into the JG101 strain. Cells were then plated on LB medium containing erythromycin (10 μg/mL) and kanamycin (10 μg/mL) to select for both BK alleles. The transformants were confirmed by PCR using primers (cheW FW/cheW RV).

To build the Mfd variants affected in Uvr recruitment, RNAP interaction, and translocation, the open reading frame of *mfd* containing base substitutions encoding each of the following single amino acid substitutions, R177A, L522R, or G977R, was synthesized by Twist Bioscience and cloned into the loading vector, pTWIST. Restriction digestion with SalI and NheI was used to subclone the *mfd*-allele from pTWIST and create the same sticky ends into the pDR111 integration vector. Competent *E. coli* cells were transformed with the *mfd*-variant plasmid as previously described (15). Transformants grown on selective plates (Amp, 100 μg/mL) were subjected to restriction digest diagnostics, and the entire plasmid was sequenced. Plasmids with the correct sequence were transformed into competent YB9801 cells and plated on LB agar containing 100 μg/mL spectinomycin to select for chromosomal integration into the *amyE* locus and 10 μg/mL tetracycline. PCR confirmed this integration using primers (Pspac_F/ lacI_R).

To construct the *hag* variants for flagella imaging, the pDR111 integration vector expressing the *hag* (T209C) allele, gifted by the Kearns lab (27), was transformed into the YB955, YB980, and JG101 strains via natural competence. Transformants were plated on LB agar containing 100 μg/mL spectinomycin for chromosomal integration and PCR-confirmed integration at the *amyE* locus using primers (Pspac_F/ lacI_R). The full list of all strains used in this study and primers is provided in the Supplemental Information (Table S1).

### Growth assays in rich medium

A single colony was used to start a 2-mL PAB overnight culture. To start the cultures for growth assays, the OD_600_ of each overnight culture was measured. One milliliter of cells was diluted to an OD_600_ of 0.4 and inoculated into a sterile flask containing 25 mL of fresh PAB. The OD_600_ was measured every 0.5 h for 6 h. Lag time was calculated by fitting OD_600_ growth curves to the Baranyi–Roberts model using nonlinear regression, from which the maximum growth rate (μ) was calculated using GraphPad Prism for three independent experiments.

### UV sensitivity assay

One milliliter of the overnight culture was inoculated into 25 mL of PAB medium. Cells were grown to an OD₆₀₀ of 1 (early stationary phase), and 10 mL of culture was harvested, washed, and resuspended in PBS (137 mM NaCl, 2.7 mM KCl, 10 mM Na₂HPO₄, and 1.8 mM KH₂PO₄, pH 7.0). One milliliter of cells was exposed to 75 J UV-C radiation (9). Colony-forming unit (CFU) counts before and after UV treatment were used to determine the percentage survival across three biological and two technical replicates.

### Read mapping

BAM files from previous RNA sequence alignments and differential gene expression analyses were retrieved to generate a raw read count matrix for genes previously identified as differentially expressed (15). To account for differences in gene length, edgeR (R package) was used to calculate Reads Per Kilobase per million mapped reads (RPKM) from the raw counts. Normalized reads were plotted against the nucleotide positions across genes. RNA-seq libraries were downloaded from the NCBI SRA database (Bioproject ID PRJNA673980).

### Quantitative real-time PCR analysis

Transcript levels were measured using the qScript One-Step RT-qPCR Kit, according to the manufacturer’s protocol (Quantabio). A total of 2 mL of T_90_ cultures was harvested, and the cell pellets were resuspended in 500 µL of RNAlater (Thermo Fisher). Total RNA was extracted using the MP FastRNA Pro Blue Kit and treated with DNAse and RNase inhibitors (Waltham, MA, USA). DNA removal was confirmed using PCR.

The relative gene expression was quantified using the 2^−ΔΔCt^ method (28). Transcript levels from the promoter-proximal (A1) and promoter-distal (A2) regions were normalized to *rnpB*, which served as an internal control. The relative A2/A1 ratio serves as a proxy for the relative levels of complete transcripts, which were calculated by dividing the 2^−ΔΔCt^ value for the A2 region by that of the A1 region, as previously reported (29, 30). Three biological and three technical replicates were performed for each condition. PCR amplification efficiency for each primer pair was assessed using a standard curve method (28), ensuring that the target gene primers had similar efficiencies to those used for the reference gene. All experiments included a no-template and no-reverse transcription (RT) control. All oligonucleotides used to quantify the expression levels in this study are listed in the supplementary materials (Table S1).

### Analysis of mRNA stability

To calculate mRNA half-life, we followed a modified version of an established protocol (31). Cultures were grown to T_90_ cells, and rifampicin (10 μg/mL) was added to the culture medium. Cells were harvested at specified time points (*t* = 0, 4, and 6 min), immediately placed on ice, and total RNA was extracted using RNA extraction kits, followed by treatment with DNase to degrade DNA and RNase inhibitors to preserve RNA. Transcript levels were measured using the qScript One-Step RT-qPCR Kit, following the manufacturer’s protocol, as mentioned above. The relative mRNA abundance at each time point was determined using the 2^−ΔCt^ method, where Ct values were normalized to the average Ct at *t* = 0 min. mRNA decay kinetics were analyzed using GraphPad Prism’s one-phase exponential decay model. To evaluate the potential effects of rifampicin treatment on mRNA stability, the integrity of the total RNA from T_90_ cells treated with and without rifampicin was measured using an Agilent 2100 Bioanalyzer. The RNA integrity number (RIN) was calculated; a value greater than 8 was considered highly stable RNA (32). Each experiment included three technical replicates and three biological replicates from independent experiments.

### Motility assay

To test for flagellum-based motility, a single colony was used to start a 2 mL PAB overnight culture with appropriate antibiotics when needed. On the following day, the OD_600_ of each overnight culture was measured. Cells were diluted to an OD_600_ of 0.1 for each strain and replicate. Ten microliters of the cell dilution was inoculated into swimming agar plates containing 20 mL LB with 0.3% agar, which was prepared fresh and dried for 5 h, as previously described (15, 26). The swimming diameter was measured 16 h after incubation at 37°C. At least three biological replicates were used for each experiment. Images were taken using an iPhone 13 (Apple, USA).

### Microscopy analysis

Fluorescence microscopy was performed using a Zeiss Axio Imager Microscope with a phase-contrast alpha Plan-Apochromat 100× objective. Alexa Fluor 488 Cs maleimide (Molecular Probes) fluorescent signals were measured (X-cite 120LED) in the range of 470–525 nm (false color green) and FM4-64 (Molecular Probes) in the range of 550–607 nm (false color red), and images were recorded using ZEN 3.3 Pro software. For fluorescence microscopy of flagella, 1 mL of cells was harvested at an OD_600_ of 0.8 and washed once in 1.0 mL of PBS. The suspension was pelleted, resuspended in 50 μL of PBS containing 5 µg/mL Alexa Fluor 488 C and maleimide (Molecular Probes), and incubated for 5 min at room temperature. The cells were then washed twice with 50 μL PBS. Membranes were stained by resuspension in 50 μL of PBS containing 5 g/mL FM4-64 (Molecular Probes), incubated for 10 min at room temperature, and then washed twice with PBS. Five microliters of the suspension was mixed with 1 μL of medium and placed on 1% agarose pads, as previously described (27).

Microscopy images were processed and analyzed using the Fiji distribution (http://fiji.sc) of ImageJ (https://imagej.net/ij/). Fluorescence was quantified using the Corrected Total Fluorescence (CTF) formula (33). The proportion of flagellated cells per image was calculated as the ratio of CTF of Alexa Fluor 488 C to the membrane signal (FM4-64), using four technical replicates and three biological replicates. To quantify flagella levels of “singlet” cells, the CTF of Alexa Fluor 488 C was measured in at least 50 single-unchained cells and normalized to cell length. Phase contrast images were obtained to calculate the percentage of “chained” cells (27). Cells with three or more chains were categorized as “chains.” Finally, the number of cell chains was divided by the total number of cells and converted to a percentage. At least 500 cells were counted for each biological replicate, resulting in a total of three replicates. Samples were observed under phase contrast at 1,000× magnification and recorded using ZEN 3.3 Pro software.

### Western blotting

Cytoplasmic protein levels of Hag and SigA were analyzed by Western Blot analysis, as described in references 34–36. Cells were grown in PAB to an OD₆₀₀ of 1 and normalized to cell density, harvested, and resuspended in lysis buffer (20 mM Tris-HCl [pH 7.0], 10 mM EDTA, 1 mg/mL lysozyme, 100 mM phenylmethylsulfonyl fluoride [PMSF]), and incubated for 30 min at 37°C. Samples were then lysed by sonication using a probe sonicator (Branson Sonifier 450, VWR) at 50% duty cycle, output control 3 setting for a total sonication time of 2 min while samples were kept on ice. 200 μL of lysate was mixed with 50 μL 4× Laemmli buffer (200 mM Tris-HCl [pH 6.8], 8% SDS, 40% glycerol, 0.04% bromophenol blue, 20% 2-mercaptoethanol).

Equal volumes of normalized and heat-denatured protein preparations were electrophoresed on 12.5% SDS-PAGE gels with a SeeBlue Plus2 Prestained Standard (Invitrogen). For Western blot analyses, Hag was probed with a 1:80,000 dilution of an anti-Hag primary antibody, and the loading control SigA was probed with a 1:80,000 dilution of an anti-SigA primary antibody (both antibodies were generous gifts from the Kearns lab). In both cases, a 1:1,000 dilution of the GE anti-rabbit IgG–horseradish peroxidase (HRP; NA9340) secondary antibody was used. All blots were imaged using the “auto expose” setting with chemiluminescence detection (Azure 500; Azure Biosystems). Densitometry was done using the lane and band detection features of the AzureSpot analysis software. The experiments were performed with two technical replicates and two biological replicates.

### Capillary assay

Capillary assay was performed as previously described (37). A single colony was grown overnight in 2 mL of tryptone minimal medium (1% tryptone, 0.5% NaCl, 0.14 mM CaCl_2_, 0.20 mM MgCl_2_, and 0.01 mM MnCl_2_). The cultures were grown in fresh tryptone minimal medium to an OD_600_ of 0.6 (mid-exponential) at 37°C and 250 rpm shaking. At this point, 50 μL of GL solution (5% glycerol and 0.5 M sodium lactate) was added to the culture, and the cells were incubated for an additional 15 min. Cells were then washed twice with chemotaxis buffer (0.01 M K_3_PO_4_, 0.14 mM CaCl_2_, 0.3 mM (NH_2_)_2_SO_4_, 0.1 mM EDTA, 5 mM sodium lactate, 0.05% glycerol, and pH 7.0) and incubated for an additional 20 min (at 37°C, 250 rpm shaking) to ensure that the cells were motile. Cells were diluted to an OD_600_ of 0.002 in chemotaxis buffer and then aliquoted into 0.1 mL ponds with chemotaxis buffer at pH levels of 6, 7, or 8 on a slide warmer at 37°C for 5 min. A capillary tube filled with 20 μL of the attractant (pH 7) was inserted into the pond at 37°C for 1 h. Cells that migrated into capillaries were harvested and transferred to minimal tryptone plates. The plates were incubated at 37°C for 16 h, and the number of colonies was counted and normalized to the control pond condition (pH 7). The experiments were performed with two technical replicates and three biological replicates.

### Identification of non-B DNA motifs

We used the web-based tools QGRS Mapper (38) and nBMST (39) to identify potential non-B DNA motifs within the nucleotide sequences of the genes of interest. Predicted motifs were analyzed using ViennaRNA Package 2.0 (40) to calculate the minimum free energy (MFE) structures. Only motifs with an MFE below −5 kcal/mol have been reported, as such values indicate stable, physiologically relevant secondary structures (41, 42).

### Statistical analysis

We used one-way analysis of variance (ANOVA) with the least significant difference (LSD) test and Student’s t-test, when appropriate, via R Studio programming. Mean comparisons were conducted in pairwise combinations, and statistically significant differences (*P* ≤ 0.05) between any two means were denoted by different letters or *. We assigned “a” to the means that were not significantly different from the mean with the highest value, “b” to means that were different from the “a” group, etc.

## RESULTS

### Mfd controls the completion of full-length transcripts at select motility loci in the stationary phase of *B. subtilis*

**Fig 1:**
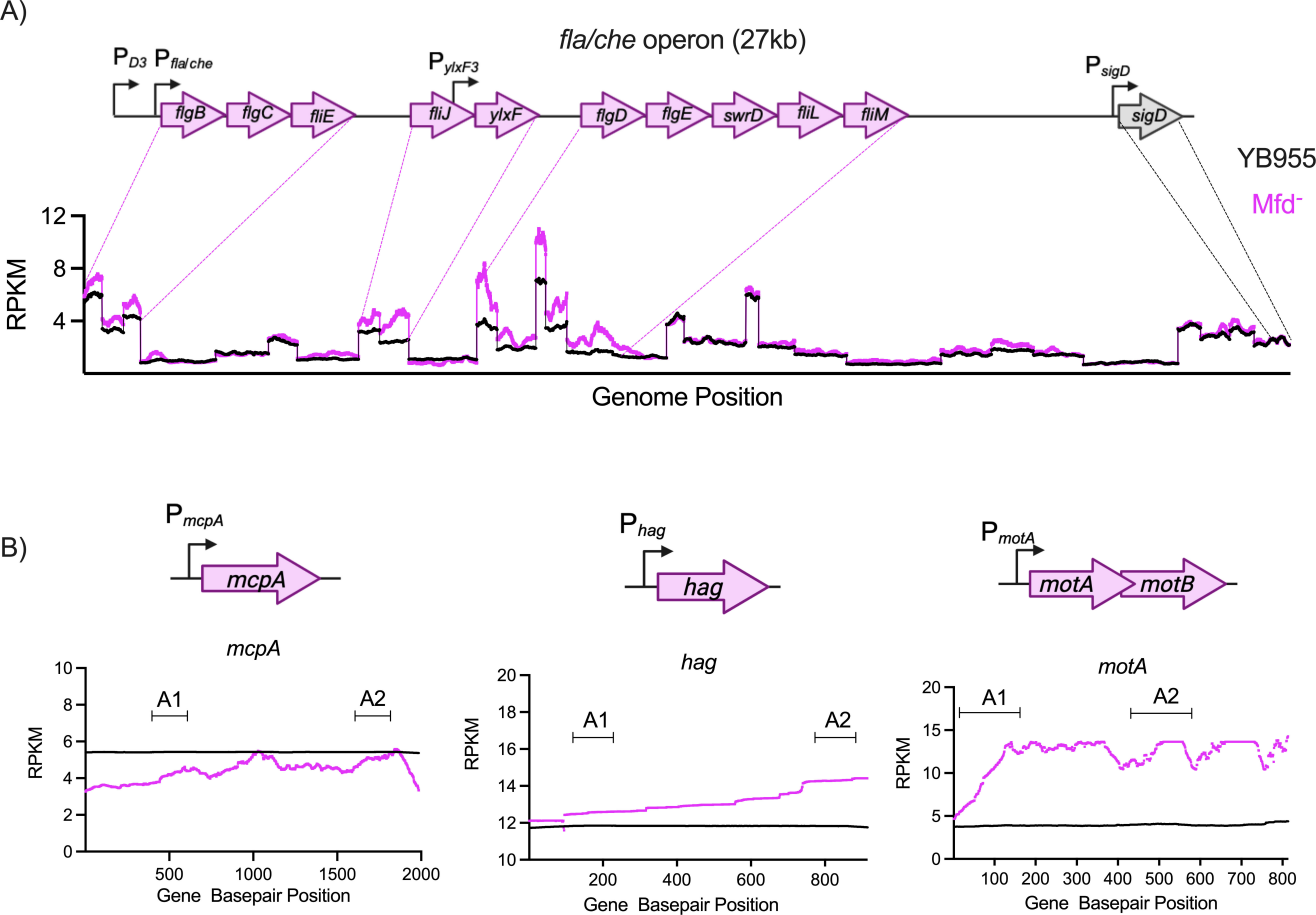
Loss of Mfd alters read coverage of motility genes in the stationary phase of *Bacillus subtilis*. RNA-seq coverage tracks are shown for the parental *B. subtilis* YB955 (black) and the Mfd-deficient strains (magenta), both grown to the stationary phase in rich medium. The RPKM mapped read values were plotted across each nucleotide position for (**A**) the *fla/che* operon and (**B**) SigD-dependent genes associated with motility and chemotaxis. Gene names are indicated with open arrows above the x-axis. Bent black arrows represent promoters. Magenta open arrows highlight motility-related genes that were differentially expressed in the Mfd-deficient strain. Not all genes in the operon are shown. Genes and operons are not drawn to scale. Regions corresponding to the promoter-proximal (A1) and promoter-distal (A2) RT-qPCR amplicons are shown aligned to the coverage tracks.

To evaluate the impact of Mfd on the transcription profile of motility genes, we calculated the RPKM for both parental and Mfd-deficient strains (Fig. 1). The genes in the 5′ region of the *fla/che* operon exhibited higher read coverage in Mfd-deficient cells than in parental *B. subtilis (*Fig. 1A). Interestingly, the read coverage in the distal or 3′ region of the *fla/che* operon, including *sigD*, was not affected by Mfd. We also observed differential read coverage, even within the open reading frame (ORF) of genes corresponding to the *fla/che* operon (Fig. S1A) and other genes required for motility (Fig. 1B). Notably, the Mfd^−^ cells show a fourfold increase in the read coverage of the *motA* gene compared to the parent. The *motA* gene encodes the flagellar stator unit, which is critical for generating torque during flagellar rotation (19, 43).

**Fig 2:**
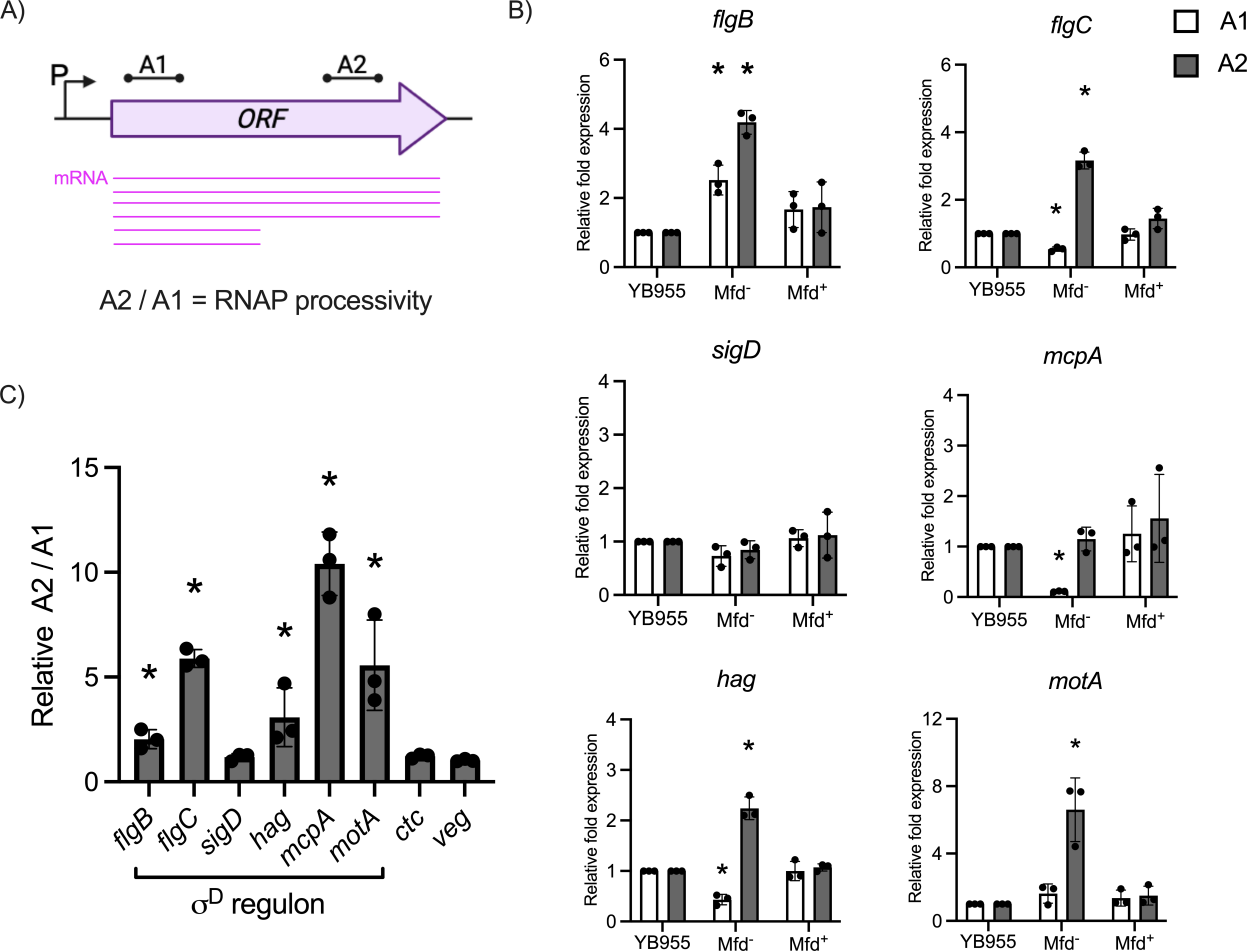
Mfd modulates loci-specific transcript levels of motility genes in stationary-phase *B. subtilis*. (**A**) Schematic of RT-qPCR amplicons targeting promoter-proximal (A1) and promoter-distal (A2) regions. An increase in the relative A2/A1 ratio reflects increased RNA polymerase elongation efficiency, as it indicates a greater abundance of promoter-distal transcripts (A2) relative to promoter-proximal (A1) ones. (**B**) Relative quantification of region-specific mRNA levels for selected motility genes in the Mfd-deficient (Mfd^−^) and complemented (Mfd^+^) strains measured by RT-qPCR. Relative expression was adjusted to the expression of the *rnpB* gene (unaffected by Mfd as shown by Martin et al. [15]; see Quantitative Real-Time PCR Analysis in Materials and Methods). Gene names are indicated above the x-axis. (**C**) Gene-specific relative A2/A1 ratios in the Mfd-deficient strain. Motility genes controlled by the alternative sigma-factor D (σ^D^) are grouped. (*) corresponds to *P* < 0.05 by one-way ANOVA with LSD test.

Because regions in the ORF with high read density often correspond to areas where RNAP pauses or stalls (14, 44), we hypothesized that the resulting profile represents regions where Mfd interacts with stalled or paused elongation complexes to control the production of full-length transcripts. To test this idea, we designed primers with similar amplification efficiency and amplicon size to measure the relative expression levels of the 5′ end (A1) and the 3′ (A2) transcripts of select genes within the σ^D^-motility regulon (Fig. 2A)*,* with the expectation that transcripts that contain the A2 region correspond to longer or full-length RNA molecules (29, 30). We measured the relative expression levels of region-specific transcripts in the parental, Mfd-deficient, and Mfd-complemented strains grown to the stationary phase. We observed that the loss of Mfd led to at least a twofold increase in promoter-distal or A2 transcripts (Fig. 2B), consistent with our transcriptomics (Fig. 1; Fig. S1A). Notably, the Mfd-complemented strain restored transcript levels to those observed in the parental *B. subtilis* strain, supporting the hypothesis that Mfd controls the completion of transcripts.

We then calculated the relative ratio of A2/A1 transcripts, which has been used as a proxy to measure the relative levels of full-length transcripts, effectively reflecting the efficiency with which RNAP progresses through the gene body *in vivo* (29, 30). The Mfd-deficient strain showed significant increases in the A2/A1 ratio in *flgB*, *flgC*, *hagA*, *mcpA*, and *motA* compared to the parental strain, suggesting enhanced transcript completion at specific motility loci without influencing *sigD* transcription (Fig. 2C). Interestingly, we found that the loss of Mfd resulted in reduced levels of promoter-proximal (A1) transcripts for *flgC*, *mcpA*, and *hag*, while the relative A2/A1 transcript ratio increased. This pattern suggests altered early transcriptional dynamics. As a control, we analyzed σ^D^-independent genes (*ctc*, *veg*). We found that the loss of Mfd did not affect read coverage (Fig. S1B) and A2/A1 ratios (Fig. 2C*;* Fig. S1C). We concluded that Mfd mediates premature transcription termination in a locus-specific manner, selectively influencing the expression of specific motility genes.

### Mfd influences transcription without affecting mRNA half-life

To determine whether the increased transcription observed in Mfd-deficient cells could be related to mRNA stability or decay, we measured the effects of Mfd on mRNA half-life. mRNA stability was assessed by treating stationary-phase *B. subtilis* cultures with rifampicin, an antibiotic that inhibits transcription initiation (45). Total RNA was extracted before (*t* = 0 min) and at subsequent time points (*t* = 4 and 6 min) following the addition of rifampicin. Using the same primers from our RT-qPCR analysis, we quantified mRNA levels using the 2^−Δ^ method, normalizing values to pretreatment levels (*t* = 0 min) (31). The resulting decay curves were analyzed using a one-phase exponential decay model to calculate the mRNA half-lives. Our results showed that the absence of Mfd did not affect the half-life of any region-specific transcript (Fig. S2A). To rule out indirect effects of rifampicin on RNA quality, we assessed RNA integrity and found no impact (Fig. S2B). Collectively, these results indicate that the effects of Mfd on transcription are independent of mRNA stability.

### Mfd variants are sensitized to UV radiation and defective in TCR function

To assess the function of Mfd’s structural features on the expression of motility genes, we constructed three Mfd variants, each carrying a single amino substitution in key functional domains: the translocase domain (TRG), the RNA polymerase-interacting domain (RID), and the Uvr module (Uvr) (Fig. S3A). We performed a UV sensitivity assay to assess the functional impact of the L522R, R177A, and R962A mutations, which map to the RID, Uvr, and TRG domains, respectively, as predicted by studies in the *E. coli* homolog (3, 46, 47). UV radiation induces bulky DNA lesions, such as thymine dimers, which stall RNAP and trigger TCR to selectively remove damage from the template strand (3). All three Mfd variants exhibited increased UV sensitivity, comparable to that of the Mfd-null strain (Fig. S3B), independent of growth, as we found no difference in the growth rate when performing the growth assay (Fig. S3C). Additionally, there were no indirect effects associated with the pDR111 integrative shuttle vector expressing the *mfd* alleles. Based on these findings, we conclude that all three Mfd variants are functionally impaired in their respective domains for carrying out TCR.

### Mfd suppresses transcript completion through interaction with RNAP

**Fig 3:**
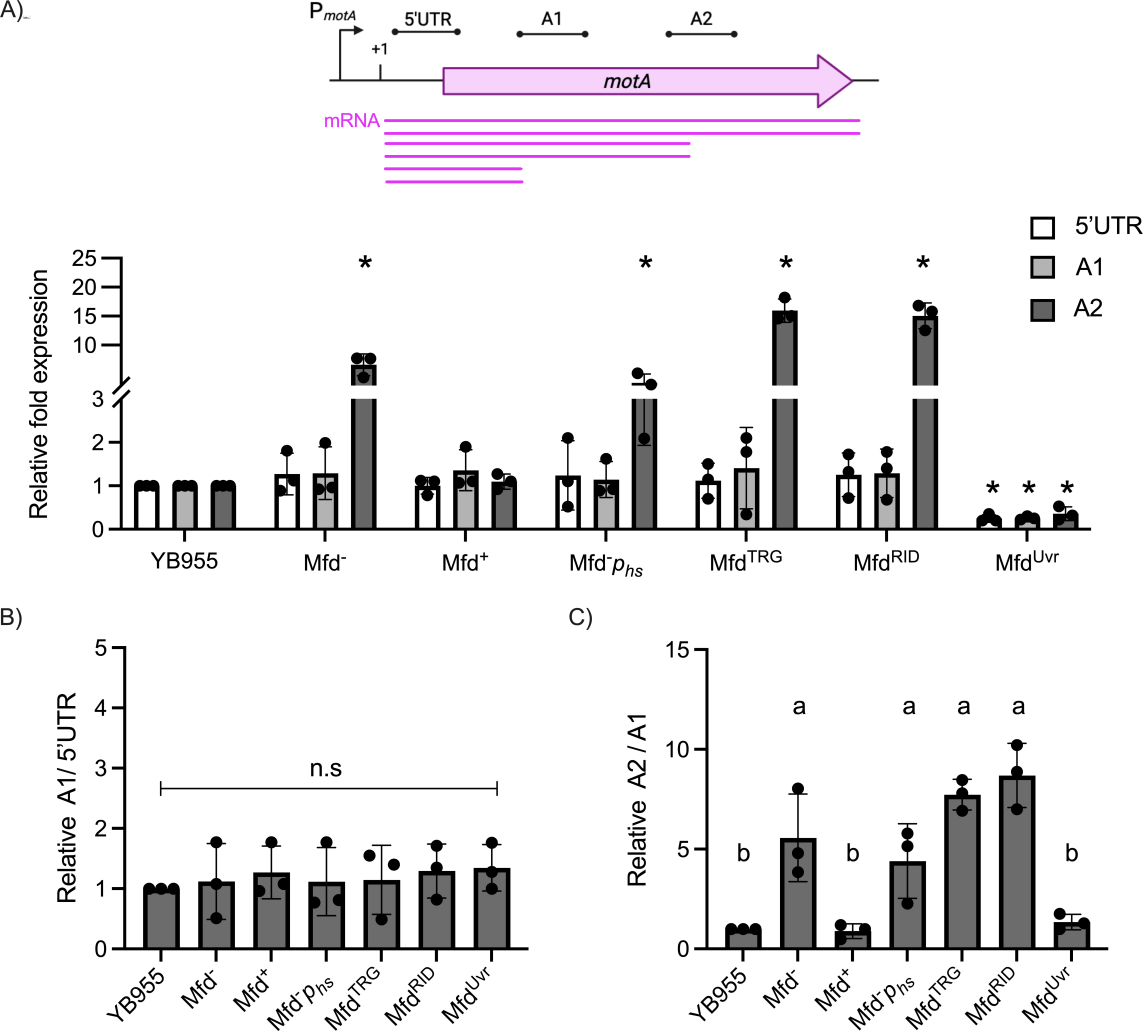
Mfd translocation and RNA polymerase association—but not UvrA recruitment—suppress the completion of *motA* transcripts. (**A**) RT-qPCR was used to quantify the relative transcript abundance in three regions of the *motA* gene: the 5′ UTR, the promoter-proximal (A1) site, and the promoter-distal (A2) site. (**B and C**) Ratios of 5′ UTR/A1 and A2/A1 were calculated for each strain and normalized to the parental *B. subtilis* YB955 strain. An increase in the 5′ UTR/A1 or A2/A1 ratio indicates enhanced RNA polymerase elongation efficiency in those regions, reflecting a higher abundance of promoter-distal transcripts relative to promoter-proximal ones. The levels of mRNA were measured for the Mfd-deficient (Mfd^−^P_hs_: Mfd mutant containing an empty cloning vector with an IPTG-inducible promoter integrated into the *amyE* chromosomal locus), complemented strain (Mfd^+^: Mfd mutant containing an IPTG-inducible *mfd* gene in single copy integrated into the *amyE* chromosomal locus), as well as for mutant alleles of *mfd,* including G977R (Mfd^TRG^), L522R (Mfd^RID^), and R177A (Mfd^Uvr^). The Mfd-deficient strain with the empty integrative expression vector included as a control was also examined. Dots correspond to biological replicates, and bars correspond to standard deviation. Lowercase letters denote differences between means (LSD test), (*) correspond to significance at *P* < 0.05, and “n.s.” denotes non-significant by one-way ANOVA.

To determine the structural features of Mfd that influence the transcription dynamics of motility genes in stationary-phase *B. subtilis*, we measured the influence of Mfd’s specific domains on the relative expression levels of the 5′ untranslated region (UTR), A1, and A2 *motA* transcripts. We found that the loss of Mfd does not affect the relative levels of transcripts containing the 5′ UTR and A1, as indicated by the A1/5′ UTR ratio (Fig. 3A and B), showing that Mfd does not influence RNAP’s ability to transcribe these regions of the gene. These results support our hypothesis that Mfd influences the transcription of coding sequences. Disruption of Mfd translocation or RNAP interaction resulted in a fivefold increase in the A2/A1 ratio of *motA* transcripts relative to the parental strain (Fig. 3A and C). Since promoter-distal transcripts reflect longer, more full-length RNA molecules, their increase suggests that RNAP more frequently completes transcription through the *motA* gene in the absence of Mfd translocation and RNAP association. Reintroduction of wild-type Mfd restored the A2/A1 transcript ratios to parental levels, with no effect from the expression vector. Disrupting the Uvr module led to a reduction in overall *motA* transcript levels relative to the parental strain (Fig. 3A) but did not affect the relative ratio of A2/A1 transcripts (Fig. 3C). These findings suggest that the recruitment of UvrA is not required for Mfd to coordinate RNAP progression, which is consistent with previous *in vitro* studies showing that Mfd can mediate the release of nascent or incomplete transcripts independently of UvrA recruitment (47). We conclude that Mfd primarily functions during transcriptional elongation and propose that Mfd-mediated suppression of nascent transcripts is an important mechanism controlling the expression of the motility regulon in stationary-phase cells.

### Potential role of Mfd-mediated UvrA recruitment in RNAP recycling

The Mfd variant deficient in binding to UvrA exhibited reduced *motA* mRNA expression (Fig. 3A), but an unaffected A2/A1 ratio of *motA* transcripts (Fig. 3C). One possible explanation for the observed A2/A1 results in the Uvr-defective variant is changes in transcription initiation, which prompted us to explore the mechanism driving this outcome. Single-molecule reconstruction studies have demonstrated that loss of UvrA delays dissociation of the Mfd:RNAP complex during transcription (48, 49). Based on these observations, we hypothesized that impaired Mfd-mediated UvrA recruitment might reduce *motA* transcript levels by hindering RNAP recycling, thereby limiting the pool of RNAP available to initiate new rounds of transcription. We tested this idea by measuring the activity of two promoters with different activities. The *veg* promoter in *B. subtilis* has been established as a strong constitutive promoter (50). In contrast, the promoter of *ctc* is condition-dependent (51, 52), and its activity is much lower than that of the strong constitutive *veg,* as suggested by the substantial differences in RPKM (Fig. S1B). Therefore, we assessed the effect of UvrA recruitment on the transcription of *veg* and *ctc* as benchmarks for RNAP recycling efficiency. Disruption of the Uvr module of Mfd did not affect *veg* transcription (Fig. S4B). In contrast, the same mutant showed reduced transcript levels in both the promoter-proximal and distal regions of *ctc*, without impairing elongation efficiency (Fig. S4C). The expression levels of *ctc* were restored to those of the parental strain when complemented with wild-type *mfd*, confirming the specificity of this defect. Together, these findings suggest that Mfd-mediated UvrA recruitment, beyond its established role in DNA repair, is required for modulating the optimal RNAP promoter activity of *motA and ctc* and may play a broader role in RNAP recycling.

### Mfd-dependent regulation of RNAP elongation reduces flagellation and promotes motility and cell chaining in *B. subtilis*

**Fig 4:**
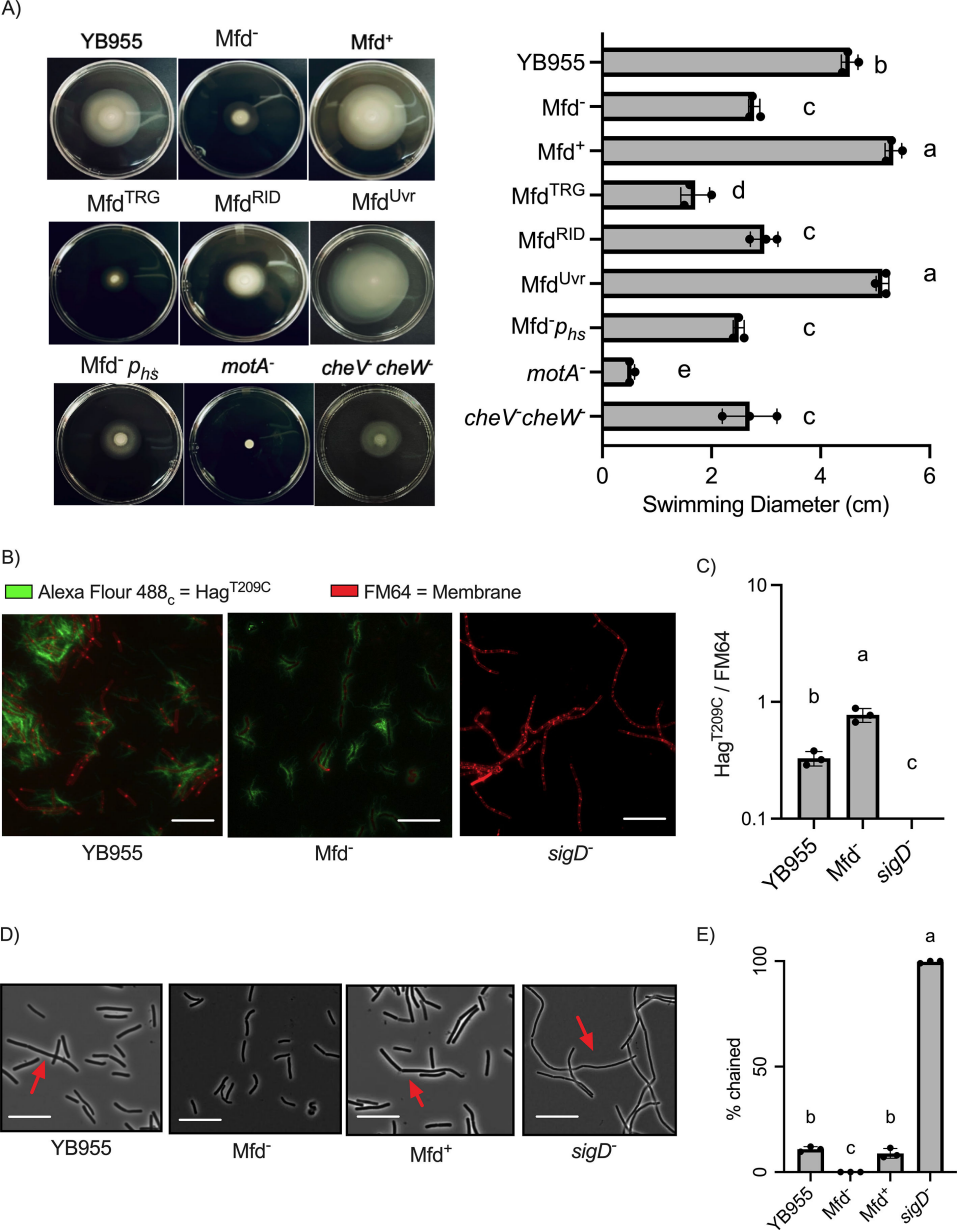
Mfd-dependent premature transcription termination reduces membrane-associated flagella and promotes efficient swimming and cell chaining. (**A**) Swimming motility assays were performed on 0.3% agar plates, and swimming diameters were measured after 16 h. Strains tested include the parental YB955, the Mfd-deficient, complemented strain (Mfd^+^: Mfd mutant containing an IPTG-inducible *mfd* gene in single copy integrated into the *amyE* chromosomal locus), as well as mutant alleles of *mfd,* including G977R (Mfd^TRG^), L522R (Mfd^RID^), and R177A (Mfd^Uvr^). Controls include the Mfd-deficient strain carrying an empty integrative vector, a *motA*-deficient strain, and a double *cheV cheW* mutant. Dots correspond to biological replicates, and bars correspond to standard deviation. (**B**) Fluorescence microscopy was performed on Hag^T209C^ variants of YB955, Mfd, and the SigD-deficient strain. Membrane is stained with FM4-64 (false colored red), and flagella (Hag^T209C^) are stained with Alexa Fluor 488 C5 maleimide (false colored green). Scale bar (8 μm) included. (**C**) Quantification of Hag^T209C^ fluorescence levels normalized to FM4-64 fluorescence (s*ee* Materials and Methods) performed on Hag^T209C^ variants of YB955, Mfd^−^, and the SigD-deficient strain. Membrane is stained with FM4-64 (false colored red), and flagella (Hag^T209C^) are stained with Alexa Fluor 488 C5 maleimide (false colored green). (**D**) Phase contrast images showing *B. subtilis* populations consist of two distinct morphological forms: single or double cells and chains of three or more cells (red arrows). These were categorized as “singlets/doublets” and “chains,” respectively. Scale bar (4 μm) included. (**E**) The number of cells in each category was divided by the total number of observed cells and converted to percentages. For each strain, at least 500 cells were counted across multiple fields of view. Dots represent biological replicates; error bars show standard deviation. Lower case letters denote differences between means (LSD test).

We demonstrated that the structural features of Mfd control transcriptional progression at the *motA* gene in the absence of exogenous DNA damage (Fig. 3). We investigated which Mfd domain was important to control the ability of RNAP to complete a transcript, which correlates with the phenotype, by inoculating cells on 0.3% agar plates (15, 26). As a negative control, we included a *motA*-deficient strain that was non-motile and remained at the inoculation site (Fig. 4A). We found that inactivating *mfd* or impairing Mfd’s ability to translocate or bind to RNAP significantly reduced swimming motility, nearly halving the swim diameter compared to the parental strain (Fig. 4A). Notably, restoring Mfd rescued motility, likely by reestablishing Mfd-mediated regulation of motility genes or broader transcriptional networks, which is consistent with previous findings (6, 15). Interestingly, disruption of the Uvr module of Mfd resulted in an unexpected increase in swimming.

In the absence of Mfd, transcription of the *hag* gene, which encodes flagellin, the major structural component of the flagellar filament (19), increased (Fig. 1 and 2). We hypothesized that increased *hag* expression contributes to motility defects observed in the Mfd mutant by affecting flagellar abundance or morphology. To test this, we generated Hag^T209C^ variants that enabled fluorescent labeling of the flagella using Alexa Fluor 488_C_ and cell membrane using FM64 during the early stationary phase (Fig. 4B) (27). Mfd-deficient cells exhibited an increase in the proportion of flagellated cells as determined by quantifying Alexa Fluor 488_C_ fluorescent levels normalized to FM64 (Fig. 4B and C). Notably, when calculating Hag^T209C^ fluorescent levels at the single-cell level, loss of Mfd leads to an increase in this ratio (Fig. 4B; Fig. S5A), suggesting that Mfd suppresses hyper-flagellation. Consistent with these findings, loss of Mfd resulted in increased cytoplasmic levels of Hag, as determined by Western blot using anti-Hag antibodies (Fig. S5B). As a loading control, we detected cytoplasmic protein levels of SigA using anti-SigA antibodies. As a negative control, Hag and SigA protein levels were also measured in a SigD-deficient strain.

Interestingly, loss of Mfd also eliminated the filamentous (chained) subpopulation, as reflected by the decrease in the ratio of single to chained cells and the Mfd complement strain restored the chained sub-population (Fig. 4D and E). As a control, we included a *sigD*-deficient strain that lacks Hag and contains primarily chained cells (Fig. 4B through E; Fig. S5A and B). Laboratory strains of *B. subtilis* (e.g., 168 and YB955) typically exist as long chains of sessile cells during exponential growth, with motile single cells or doublets becoming more abundant as cultures enter the stationary phase (27). These findings suggest that the Mfd mutant either transitions prematurely from a filamentous to single-cell state or requires Mfd to maintain the filamentous subpopulation.

### Mfd is essential to acid sensing in *B. subtilis*

Successful bacterial swimming requires a functional flagellar apparatus, sufficient energy, and an intact cell envelope (19). However, effective navigation also depends on chemotaxis, the ability to move toward attractants or away from repellents by altering flagellar rotation in response to chemical gradients (53, 54). This navigation system relies on chemoreceptors, such as methyl-accepting chemotaxis proteins (MCPs), as well as signal transduction components and feedback mechanisms that enable biased movement toward favorable environments (54–56). For example, the *cheV^−^ cheW^−^* double mutant, which lacks the key coupling proteins required for signal transmission (54, 56), exhibits reduced swimming, similar to the Mfd-deficient strain (Fig. 4A). Notably, our previous transcriptomic analysis revealed increased expression of multiple MCP genes [*mcpB* Log₂FC: 2.664; *mcpC* Log₂FC: 3.343; *tlpB* Log₂FC: 2.643; *tlpC* Log₂FC: 3.07] in Mfd^−^ cells (15). Interestingly, although Fig. 1 indicates that Mfd does not affect overall expression of *mcpA*, we found that transcription of the 5′ end of this gene was diminished in the Mfd^−^ cells (Fig. 2). This result suggests that Mfd is important for the RNAP to progress through *mcpA* (Fig. 2). McpA has been previously shown to be the principal MCP mediating alkaline sensing in *B. subtilis* (37). These observations prompted us to investigate whether the dysregulation of chemotaxis-associated genes resulted in pH taxis defects.

**Fig 5:**
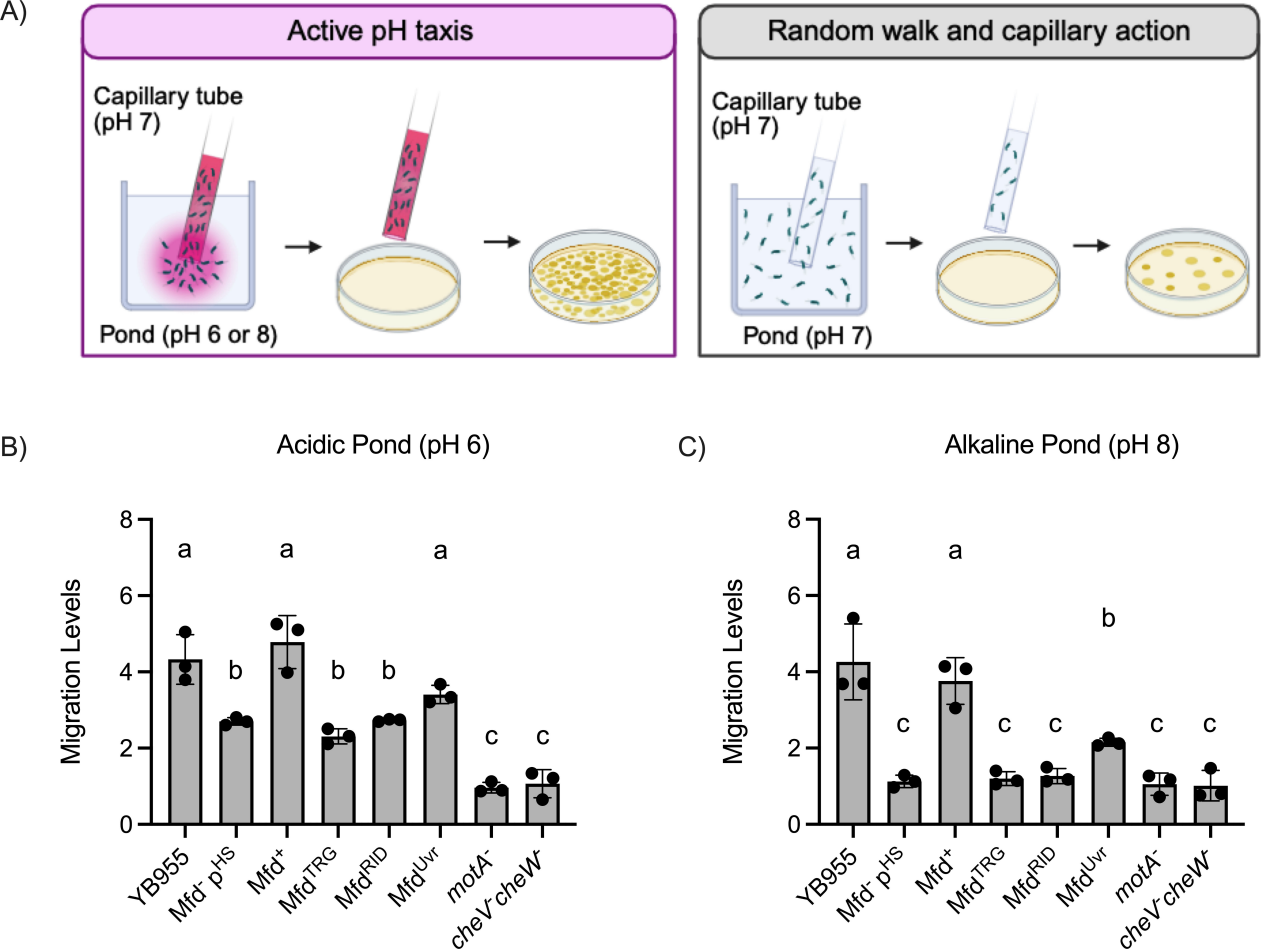
Differential requirement for Mfd in pH taxis in *B. subtilis*. (**A**) Schematic of the capillary-based pH taxis assay. Cells were inoculated into liquid “ponds” adjusted to either acidic (pH 6) or alkaline (pH 8) conditions. A capillary tube containing a neutral pH buffer (pH 7) was inserted into each pond. Cells responding to the pH gradient preferentially migrate toward the capillary, which acts as an attractant. In the control condition, cells were inoculated into a pond at neutral pH (pH 7), thereby eliminating the presence of a gradient; any cells entering the capillary under these conditions represent movement by random walk or capillary action. Cells from the capillary were recovered and plated on agar to quantify the number of CFUs, representing the cells that migrated. Migration levels are expressed as the ratio of CFUs in acidic (**B**) or alkaline (**C**) conditions and normalized to the control condition (pH 7). The strains tested include the parent YB955, Mfd-deficient, complemented (Mfd^+^: Mfd mutant containing an IPTG-inducible *mfd* gene in single copy integrated into the *amyE* chromosomal locus), as well as mutant alleles of *mfd,* G977R (Mfd^TRG^), L522R (Mfd^RID^), and R177A (Mfd^Uvr^). Controls include a *motA*-deficient strain and a double *cheV cheW* mutant. Dots correspond to biological replicates, and bars correspond to standard deviation. Lower case letters denote differences between means (LSD test).

We employed a capillary-based assay (37), in which cells were inoculated in ponds at pH 6 or 8 (Fig. 5A). A capillary containing neutral pH (7) served as a control; if cells sensed and responded to pH gradients, they preferentially migrated toward the capillary. We found that the pond conditions significantly influenced the migration of the parental strain (Fig. S5C). As expected, the non-motile *motA^−^* mutant and the *cheV^−^ cheW^−^* double mutant did not respond to pond conditions. This confirms that directed migration requires flagellar-based motility and the ability to sense and respond to a pH gradient. We showed that removing Mfd, or the ability to translocate and RNAP association, leads to a twofold reduction in migration under acidic conditions (Fig. 5B; Fig. S5C). These defects in response to acid conditions could be directly related to pH taxis or attributed to the depressed ability to swim observed in the *mfd^−^* strain; the *mfd*^−^ cells showed lower migration than the parent but higher than the *cheV*^−^/*cheW*^−^ cells (Fig. 5C). We observed a different response under alkaline conditions; the *mfd*^−^ cells were abolished in the ability to migrate (they showed the same migration as the *motA*^−^ and *cheV*^−^/*cheW*^−^ cells). Our expectation was that the responses to both conditions would be diminished because of the diminished swimming in *mfd*^−^ cells (Fig. 4A). The abolished response is surprising and suggests that Mfd influences the chemotactic response to alkaline conditions independently of swimming. Complementation with the wild-type *mfd* gene restored these responses (Fig. 5C; Fig. S5C). Interestingly, the Mfd^Uvr^ exhibited a pronounced preference toward capillaries compared to the parental strain (Fig. S5C). This response could be attributed to the increased swimming ability observed in the motility assay (Fig. 4A). However, this variant was diminished in the response to alkaline conditions when compared to its acidic response (Fig. 5C). Taken together, our findings indicate that Mfd contributes to pH taxis in *B. subtilis* through mechanisms that extend beyond flagellum-dependent motility.

### Some motility genes influenced by the loss of Mfd encode non-B DNA motifs

Previous studies in *B. subtilis* demonstrated that loss of Mfd leads to increased RNAP occupancy in genes enriched with hairpin-forming sequences in nascent transcripts (14). Our laboratory further demonstrated that Mfd directly or indirectly promotes mutations in predicted non-B DNA structures, including G-rich motifs and inverted repeats (hairpins) (57). Building on this idea, we found that the loss of Mfd during the stationary phase increases the transcription of a subset of motility genes (Fig. 1 to 3), impairing swimming (Fig. 4A) and chemotaxis (Fig. 5). This led us to investigate whether intrinsic elements in the coding region of motility genes trigger RNAP pausing or stalling, thereby signaling Mfd-dependent termination.

**Fig 6:**
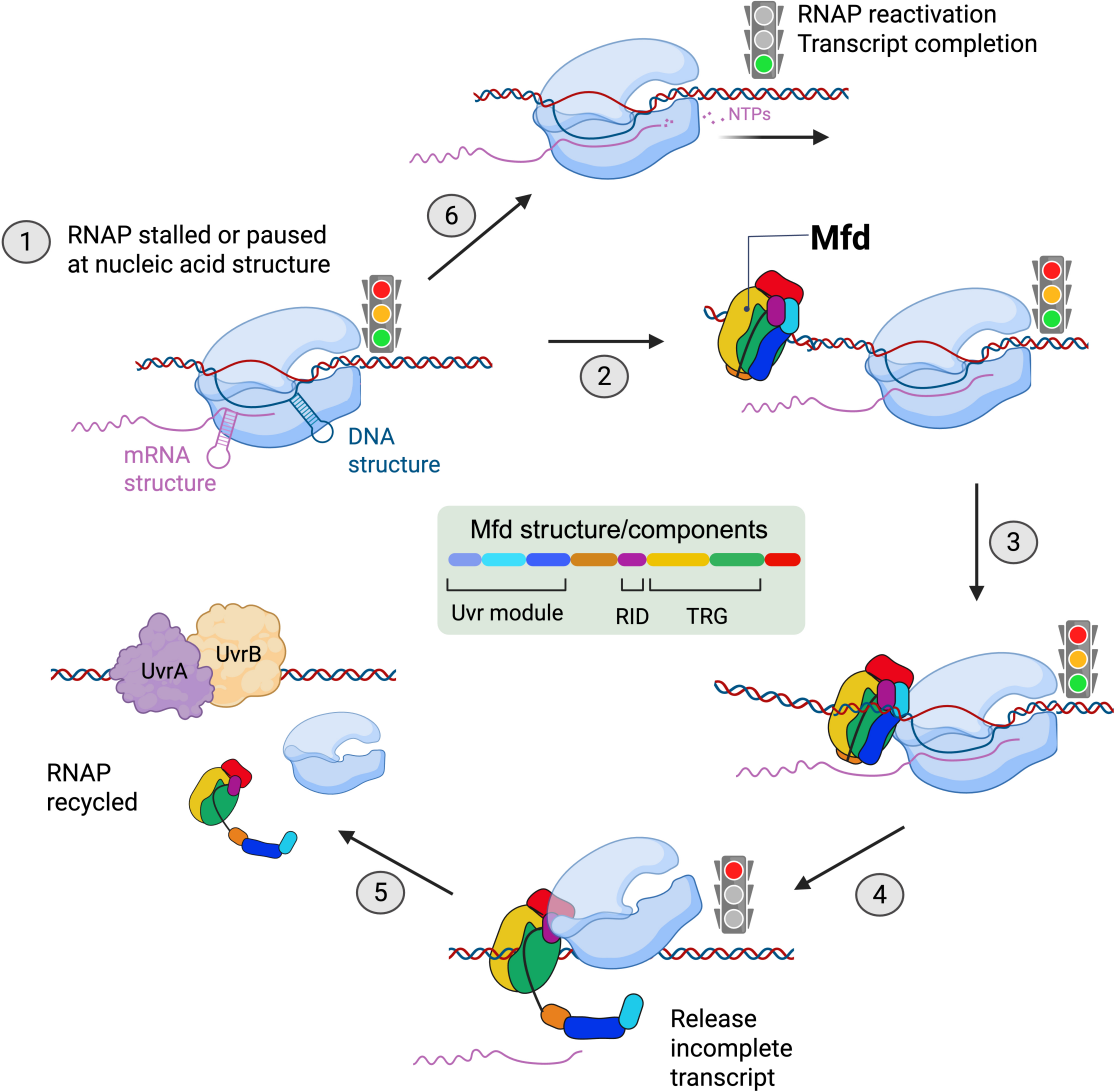
Proposed model for Mfd-mediated transcriptional control at distinct motility loci in the stationary phase in *Bacillus subtilis*. (1) During transcription elongation at distinct motility genes, the formation of secondary structures—either in the nascent mRNA near the RNA exit channel or in the template DNA ahead of the transcription bubble—can pause or stall RNAP, respectively. (2) Induced RNAP stalling or pausing acts as a regulatory signal to resolve RNAP impediment via Mfd-mediated premature transcription termination. (3) Mfd associates with DNA and uses its TRG (translocation) domain to scan for stalled or paused elongation complexes. (4) Following the direct interaction with RNAP, Mfd engages its RNA polymerase interaction domain (RID) to displace RNAP from the DNA, leading to the release of the nascent or incomplete transcript. (5) The stability of the impediment determines whether Mfd utilizes its Uvr domain to recruit the NER machinery to free the Mfd:RNAP complex from DNA. In this context, the Uvr interaction domain is required for efficient RNAP recycling. (6) In the absence of Mfd-mediated translocation or RNAP recruitment, RNAP impediment may be rescued by upstream RNAPs or destabilized nucleic acid structures, enabling transcript completion. Adapted from references 3, 13, 48, 49.

Using publicly available bioinformatics tools (see Materials and Methods), we identified non-B DNA motifs within the ORF of specific flagellar (e.g., *motA, hag*) and chemoreceptor (e.g., *mcpA*) genes (Table S2). We focused on motifs that form stable structures, such as hairpins and G-rich motifs, in either template DNA or nascent RNA, as supported by their corresponding minimum-energy structure (MFE). *In vitro* transcription and biochemical assays have demonstrated that secondary structures in nascent mRNA or DNA function as physical barriers and slow or stall RNAP progression, respectively (41, 42, 58). Thus, our findings support a model in which transcription-induced supercoiling promotes the formation of alternative DNA or mRNA structures that decrease RNAP processivity and may serve as regulatory signals that trigger Mfd-mediated transcription termination (Fig. 6). It is important to note that Mfd is also recruited to elongation complexes blocked by repressor-DNA complexes and mediates termination at those sites (16). Also, our previous genome analyses (59) indicated that only a fraction of G4-containing genes are affected by Mfd. So, we are partial to the idea that hairpins and G4s are one of a few intrinsic elements triggering Mfd-dependent transcription termination.

## DISCUSSION

### Domain-specific contributions of Mfd on transcription dynamics at motility loci

Although previous studies on Mfd’s role in transcription have primarily focused on DNA repair or logarithmic growth, this work provides the first characterization of how its distinct domains affect gene expression in stationary-phase cells, a condition marked by nutrient limitation and environmental stress. TRG and RID variants are unable to displace stalled RNAP or enhance roadblock repression *in vivo* (59, 60). Here, we demonstrate that TRG and RID are key domains required to suppress transcriptional progression of the *motA* gene (Fig. 3). Our results agree with those of others (3, 13, 48, 49); Mfd utilizes its translocase activity to scan for stalled or paused RNAPs upstream of the promoter-distal regions of motility genes (Fig. 6). Upon encountering an RNAP, the RID of Mfd engages with RNAP and terminates transcription, thereby limiting the accumulation of full-length or promoter-distal transcripts (Fig. 6). Surprisingly, we found that the TRG variant displayed reduced motility compared to the Mfd-null strain (Fig. 4A), despite no defects in growth (Fig. S3C). Previous *in vitro* studies have shown that although this variant is defective in DNA translocation, it can still be associated with the arrest of RNAPs at upstream DNA (61). They demonstrated that these interactions facilitate hybridization of the nascent RNA strand with the DNA template (R-loops), triggering DNA damage and mutagenesis *in vivo*. Therefore, we speculate that the reduced swimming observed in the TRG variant may be an indirect consequence of the induced R-loop formation.

Most molecular and structural data support an exclusive role for the Mfd Uvr module in lesion sensing and recruitment of NER. Consistent with *in vitro* results, the Uvr recruitment module was not required to suppress the completion of *motA* transcripts (Fig. 2). However, we found a potential role for the Uvr module in optimizing transcription dynamics by facilitating RNAP release and recycling, particularly at weak promoters such as *motA* (Fig. 3) or *ctc* (Fig. S4C). Moreover, we identified several motility genes influenced by the loss of Mfd that encode non-B DNA motifs, sequences capable of forming stable secondary structures that may impede RNAP progression (Table S2), suggesting that Mfd controls transcript completion at other genes. We previously showed that Mfd stimulates mutagenesis at sequences predicted to form stable G-quadruplexes and hairpin structures (57). These findings suggested that the stability of secondary nucleotide structures is a factor in the recruitment of UvrA by Mfd to resolve transcriptional events. Therefore, we propose that Mfd modulates RNAP progression at distinct motility genes in stationary-phase cells via two non-exclusive mechanisms: (i) Mfd suppresses the transcription of coding sequences at specific loci and/or (ii) Mfd recruits UvrA to promote RNAP recycling or relieve transcriptional impediments (Fig. 6). In the absence of Mfd, the secondary structures likely destabilize, resulting in transcription reactivation. Alternatively, trailing RNAPs rescue stalled complexes by promoting forward translocation and transcript completion (Fig. 6); elucidating between these models warrants further investigation. Interestingly, a previous *in vivo* study found no evidence of Mfd recruitment to motility gene loci in exponentially growing *B. subtilis* derivatives of the HM1 (JH642) parent strain (14), suggesting that the role of Mfd may be specific to a particular growth phase, stress conditions, or strain. To strengthen our findings, future studies at single-nucleotide resolution are needed to link Mfd recruitment and RNAP stalling in the stationary phase, which will be critical for fully elucidating the function of Mfd in transcription regulation and genome maintenance.

### Novel function of Mfd in pH sensing in *B. subtilis*

In *B. subtilis*, four methyl-accepting chemotaxis proteins (MCPs), McpA, McpB, TlpA, and TlpB, enable the bacterium to navigate toward neutral pH environments (37). We found that Mfd is important for RNAP to progress through the 5′ end of *mcpA (*Fig. 1 and 2)*,* and the loss of Mfd abolishes pH taxis under alkaline conditions (Fig. 5C), mirroring the phenotype observed in a double mutant lacking both *tlpA* and *mcpA* (37). Previous studies suggest that many of these MCPs are co-expressed (62). Thus, we propose that Mfd may coordinate the timing and levels of *mcpA* expression, and the disruption of this coordination may impair proper pH sensing and migration. Moreover, we demonstrated that disrupting the Uvr module did not diminish swimming (Fig. 4A) but impaired alkaline sensing (Fig. 5C). There is no substantial evidence to suggest that a pH of 8 alone directly damages DNA in *B. subtilis*. DNA remains structurally stable at this pH, and *B. subtilis* exhibits optimal growth even at pH values ranging from ~6.0 to 8.5, depending on the strain and medium (63, 64). In this context, the Uvr module appears to support transcription, rather than canonical DNA lesion repair. Future studies should examine how Mfd influences MCP protein abundance and membrane localization, both of which are critical for functional pH taxis. Finally, we showed that Mfd affects the chemotactic responses to acidity and alkalinity differently, suggesting an influence that is independent of flagellum-based motility (Fig. 4A and 5C). Therefore, it is crucial to explore the effects of Mfd on other forms of chemotactic movements that do not require flagella.

### Mfd-dependent termination of motility genes links transcriptional control to bacterial differentiation and stress adaptation

Loss of Mfd in the stationary-phase increased transcription of specific σ^D^-dependent motility genes, including the *hag* gene (Fig. 1 and 2), and correlates with an increase in both cytosolic protein levels and membrane-associated levels of flagellin (Fig. 4B and C; Fig. S5A and B). Although increased transcription of σ^D^-dependent motility genes resulted in enhanced flagellation, swimming motility (Fig. 4A), and pH taxis (Fig. 5) were reduced. These findings suggest that Mfd is required to coordinate flagellar gene expression with downstream processes such as proper flagellar assembly, motor function, or signal transduction necessary for effective migration. In contrast, the loss of Mfd–UvrA recruitment decreased *motA* expression (Fig. 3C) and enhanced swimming (Fig. 4A; Fig. S5C). This paradox between gene expression levels and phenotype suggests that Mfd functions beyond transcription-coupled repair, possibly influencing resource allocation during the stationary phase and non-proliferating conditions. Repression of flagellar genes under nutrient limitation has been shown to conserve ATP, GTP, and ribonucleotides, thereby enhancing translational efficiency (64–66). This aligns with reports that Mfd overexpression stimulates growth (15). Therefore, we propose that Mfd-dependent transcription termination contributes to stress adaptation by optimizing metabolic efficiency, which may explain why the Mfd-Uvr recruitment variant maintains motility despite having low *motA* transcript levels.

Previous studies have established a positive relationship between autolysin activity and flagella-dependent motility, but an inverse relationship with cell chaining (19, 67). Consistently, our earlier transcriptomics showed that loss of Mfd upregulated autolysin genes (*lytA* Log₂FC: 3.46; *lytB* Log₂FC: 2.37; *lytC* Log₂FC: 2.45; *lytD* Log₂FC: 2.71) (6). Here, we demonstrated that Mfd deficiency leads to an increase in the proportion of flagellated cells (Fig. 4B and C; Fig. S5A) and eliminates the filamentous (chained) subpopulation in the early stationary phase (Fig. 4D and E). However, unlike the prevailing model, this loss correlated with impaired motility (Fig. 4A). Together, these findings suggest that Mfd contributes to the epigenetic switch between motile and sessile states, independent of σ^D^ mRNA levels (Fig. 1 and 2). This function aligns with previous findings that link flagellar activity to the stress response. Disruption of MotA/MotB triggers poly-γ-glutamate overproduction, shifting cells from a motile to a protective mucoid state, a response that requires Mfd (68). Similarly, *mfd* deletion impaired sporulation (30), and we observed enhanced transcription completion at *motA* under sporulation conditions (*data not shown*). Future work examining the regulation of motility genes by Mfd at single-cell resolution will be essential to clarify its role in differentiation and stress adaptation in *B. subtilis*.

### Limitations of the study

We used RT-qPCR to measure intra-locus downstream vs. upstream (A2/A1) amplicon ratios as a primary readout of transcription elongation levels *in vivo* (29, 30). More targeted methods that can directly measure the production of nascent transcripts would strengthen our finding that Mfd coordinates transcription elongation dynamics at specific motility loci. A recent study found that the *mfd* promoter is heterogeneously expressed in *B*. subtilis (69), suggesting that Mfd levels may vary across individual cells. Our study examined the role of Mfd’s functional domains using bulk samples. Under these experimental conditions, cell-to-cell heterogeneity is lost. Consequently, bulk-level analysis may overlook or misrepresent the function of Mfd in population dynamics and cell differentiation. Lastly, we did not identify stable secondary nucleic acid structures in all motility genes affected by the loss of Mfd (e.g., *flgB* and *flgC*), suggesting that other factors may block RNAP. These could include chromosomal architecture (61) or protein-based roadblocks (16, 21), all of which warrant further investigation.

In conclusion, these findings suggest that Mfd influences gene regulation independent of TCR, potentially coordinating a shift toward stress resistance or developmental pathways in *B. subtilis*. The findings of this study are noteworthy because Mfd, along with many of the motility genes analyzed, such as *motA*, are conserved across a wide range of flagellated bacteria, including pathogenic species (70). This conservation highlights Mfd as a potential target for controlling bacterial growth, differentiation, and evolution.

## Data Availability

Research data supporting this publication are available upon request.

## SUPPLEMENTAL MATERIAL

Supplemental figures jb.00589-25-s0001.docx Figures S1 to S5.

Table S1 jb.00589-25-s0002.xlsx Primer sequences used in the study.

Table S2 jb.00589-25-s0003.xlsx DNA motifs.

ASM does not own the copyrights to Supplemental Material that may be linked to, or accessed through, an article. The authors have granted ASM a non-exclusive, world-wide license to publish the Supplemental Material files. Please contact the corresponding author directly for reuse.
