# Supplementary material for: Mfd interacts with RNA polymerase to modulate flagellum-dependent motility, chemotaxis, and population heterogeneity in the stationary phase of Bacillus subtilis
Source: J Bacteriol. 2026 May 14;208(6):e00589-25. doi: 10.1128/jb.00589-25 (PMC13277317; doi:10.1128/jb.00589-25)
Supplement: Supplemental figures — Figures S1 to S5. [file jb.00589-25-s0001.docx]

**Mfd interacts with RNA polymerase to modulate flagellum-dependent motility,**

**chemotaxis and population heterogeneity in stationary phase *Bacillus subtilis.***

**Supplemental figure& legends**

**
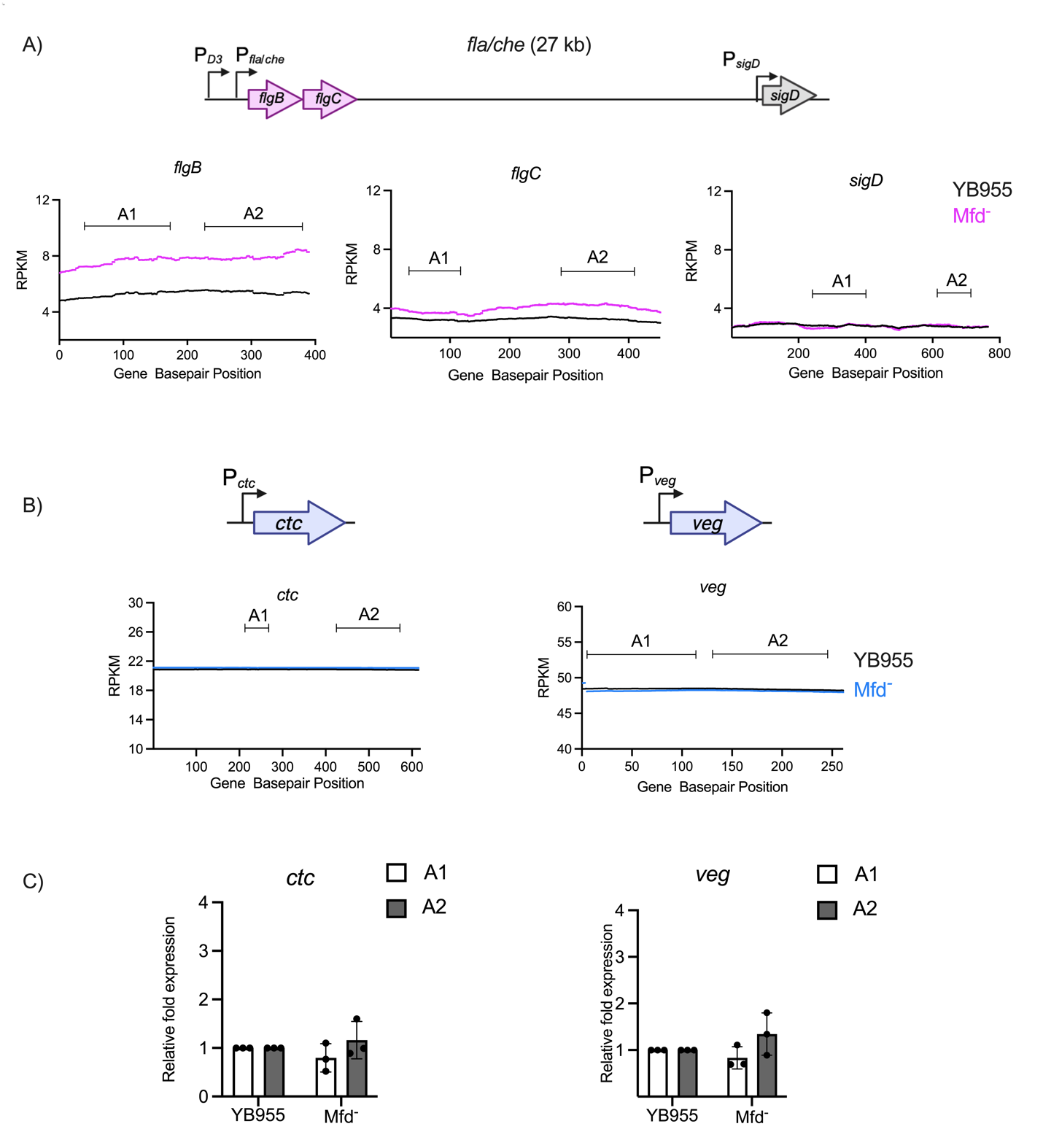
**

**Supplemental Figure 1. Locus-specific effects of Mfd on read coverage in stationary-phase *B. subtilis.*** RNA-seq coverage tracks are shown for the parental *B. subtilis* strain YB955 (black) and the Mfd-deficient strain (magenta or blue), both grown to stationary phase. RPKM (Reads Per Kilobase per Million mapped reads) values are plotted across each nucleotide position for A) genes within the *fla/che* motility operon and B) genes unrelated to motility, included as controls. Gene names are indicated with open arrows above the x-axis. Bent black arrows represent promoters. Magenta open arrows mark motility-related genes that were differentially expressed in the Mfd-deficient strain. Blue open arrows represent genes outside the motility regulon that were not determined to be differentially expressed [1]. Not all genes in the operon are shown. Genes and operons are not drawn to scale. Promoter-proximal (A1) and promoter-distal (A2) RT-qPCR amplicon regions are aligned to the corresponding coverage tracks. C) RT-qPCR quantification of promoter-proximal (A1) and promoter-distal (A2) amplicons for the non-motility genes *veg* and *ctc*. Dots correspond to biological replicates, and bars correspond to standard deviation as determined by Student’s t-test (*P* ≤ 0.05).


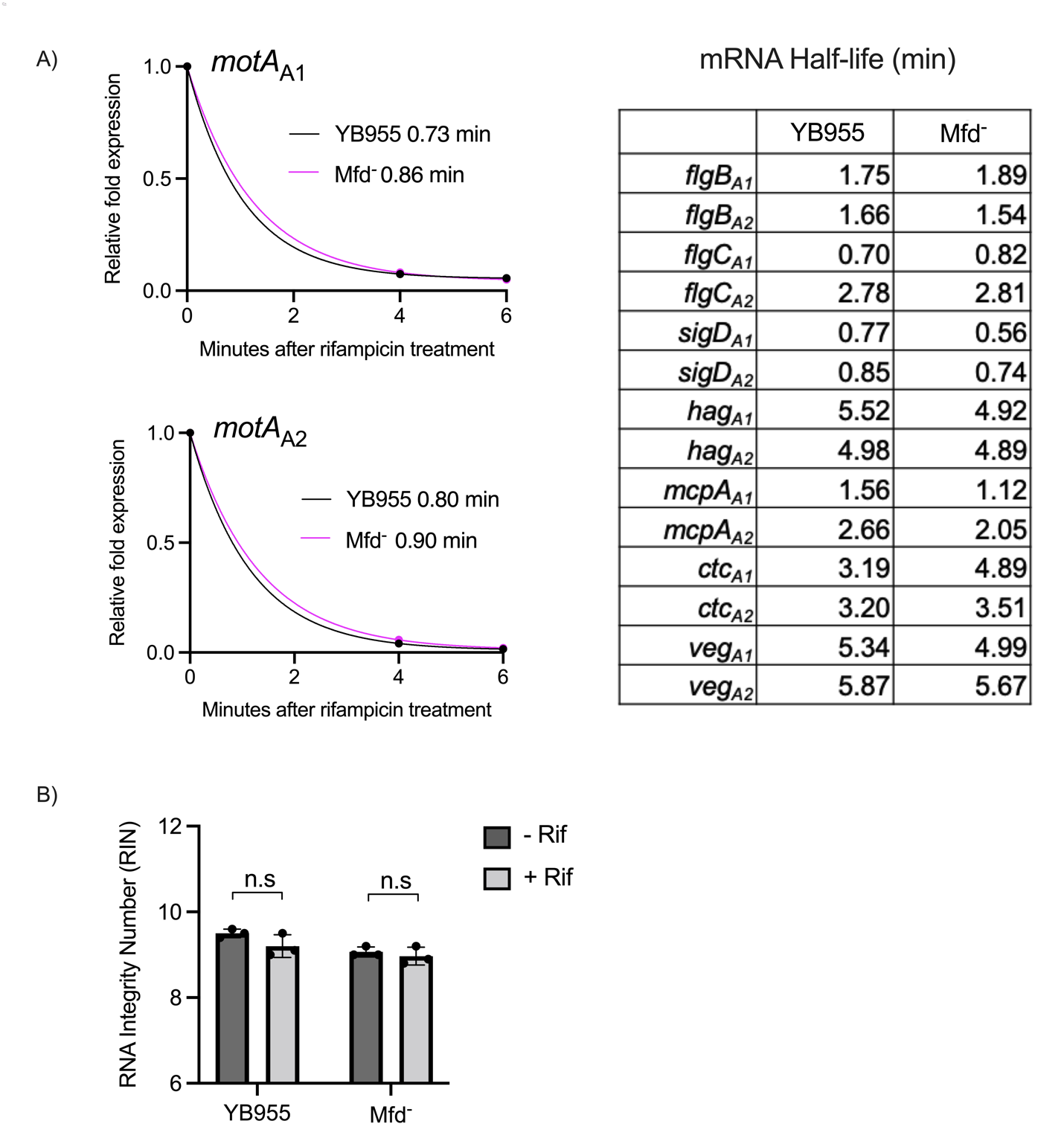


**Supplemental Figure 2. Effects of the loss of Mfd and rifampicin treatment on mRNA stability.** A) Transcript abundance was measured by RT-qPCR at multiple time points following rifampicin treatment to assess mRNA decay. Shown are the calculated mRNA half-lives and exponential decay curves for the promoter-proximal (A1) and promoter-distal (A2) RT-qPCR amplicon regions of *motA* in the parental YB955 strain (black) and the Mfd-deficient strain (magenta), both grown to stationary phase. Plotted are the relative mRNA levels at each time point following the addition of rifampicin. The one-phase exponential decay model in Prism software calculates the mRNA half-life. B) Table displaying calculated mRNA half-lives for all genes analyzed in this study. C) RNA quality was assessed by determining the RNA Integrity Number (RIN) values for total RNA extracted from the parental and Mfd-deficient strains before (-Rif) and after (+Rif) rifampicin treatment. Dots correspond to biological replicates, and bars correspond to standard deviation. "n.s." denotes non-significant differences as determined by Student’s t-test (*P* ≤ 0.05).


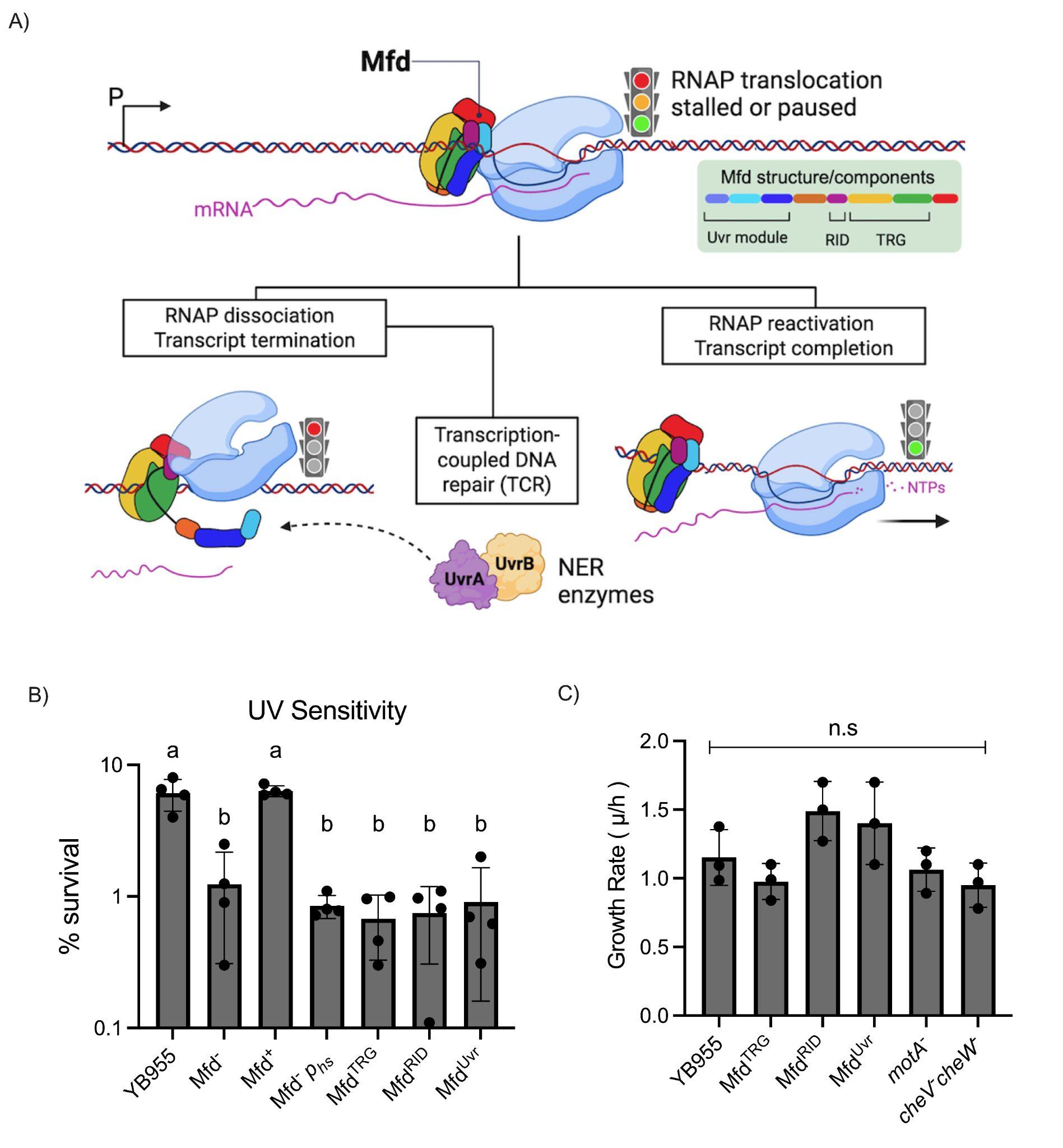


**Supplemental Figure 3. Growth profiles and UV sensitivity response of Mfd variants.** A) The figure illustrates the structure and function of Mfd on gene expression. Adapted from [2,3]. Included are the three structural domains of interest in this study: the translocase domain (TRG), RNA polymerase interaction domain (RID), and UvrA interaction module (Uvr). Mfd facilitates processing of regulatory signals that impede RNA polymerase (RNAP) elongation. Depending on the type of obstacles that interfere with RNAP, the recruitment of Mfd can reactivate the elongation complex, rescuing the progression of RNAP and facilitating the completion of full transcripts. Alternatively, Mfd can undergo significant conformational changes to dissociate the elongation complex from the DNA, thereby preventing the formation of complete RNA molecules and terminating transcription prematurely. These significant conformational changes expose the Uvr module, allowing for the direct recruitment of UvrA, a component of the nucleotide-excision repair (NER) pathway. The direct recruitment of NER initiates preferential template strand repair. B) Percent survival following UV radiation (75 J) exposure and C) growth rate of strains cultured in rich medium. The strains tested include the parent YB955, Mfd-deficient, complemented (Mfd⁺), as well as mutant alleles of *mfd,* G977R (Mfd^TRG^), L522R (Mfd^RID^), and R177A (Mfd^Uvr^). Dots correspond to biological replicates, and bars correspond to standard deviation. Lowercase letters indicate statistically significant differences between means, P < 0.05, by one-way ANOVA with LSD test.


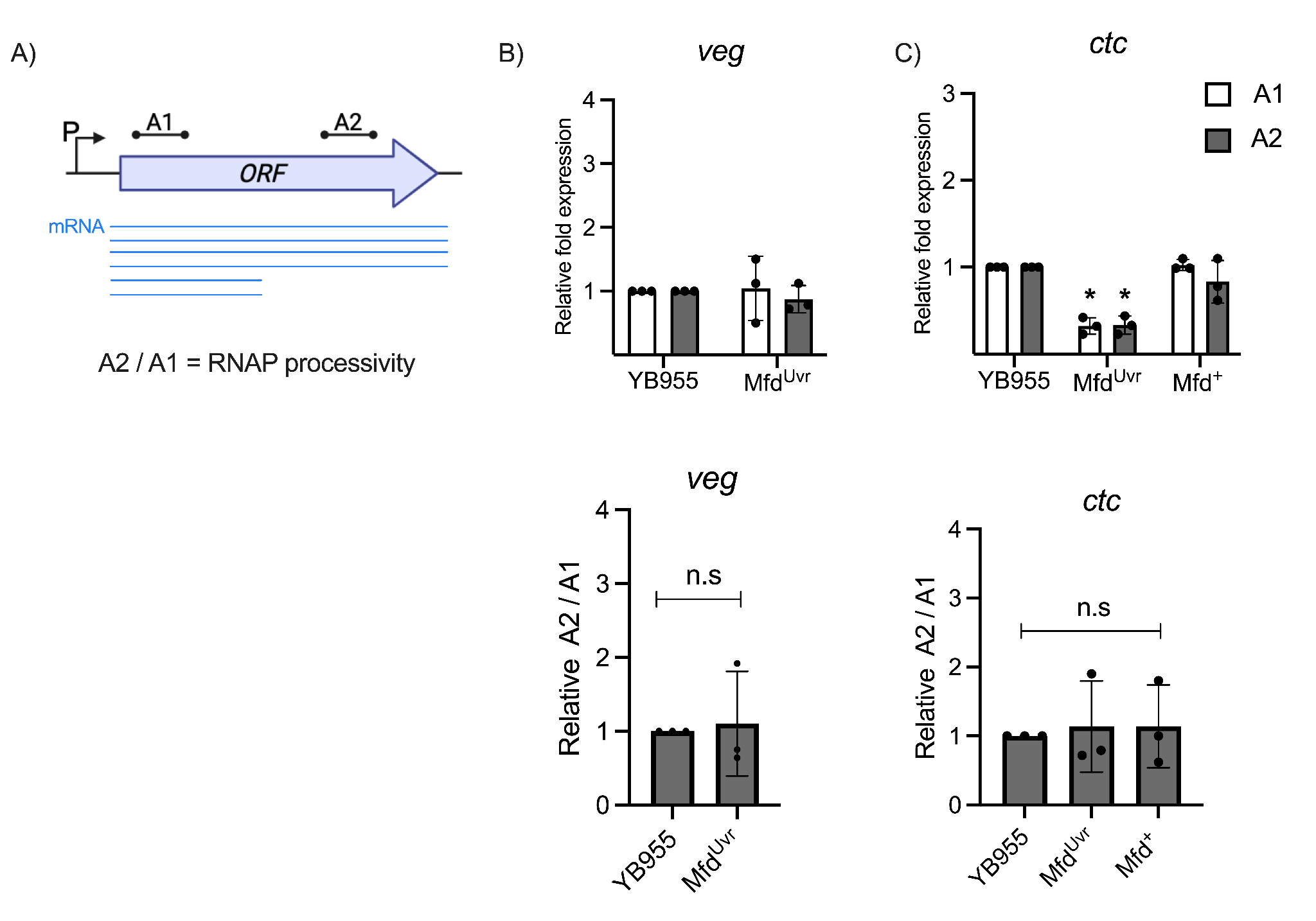


**Supplemental Figure 4. Influence of Mfd-mediated UvrA recruitment on the expression of non-motility genes in stationary phase *B. subtilis.*** RT-qPCR was used to quantify the relative transcript levels at the promoter-proximal (A1) and promoter-distal (A2) amplicon regions and to calculate the A2/A1 ratio for each strain relative to the parental *B. subtilis* YB955 strain for non-motility genes A) *veg* or B) *ctc*. A change in the relative A2/A1 ratio reflects a proportional shift in the efficiency of RNA polymerase elongation. Strains tested include the parental YB955, complemented (Mfd⁺), and *mfd* point mutant and R177A (Mfd^Uvr^). (*) corresponds to significance at P < 0.05, and "n.s." denotes non-significant by one-way ANOVA with LSD test.


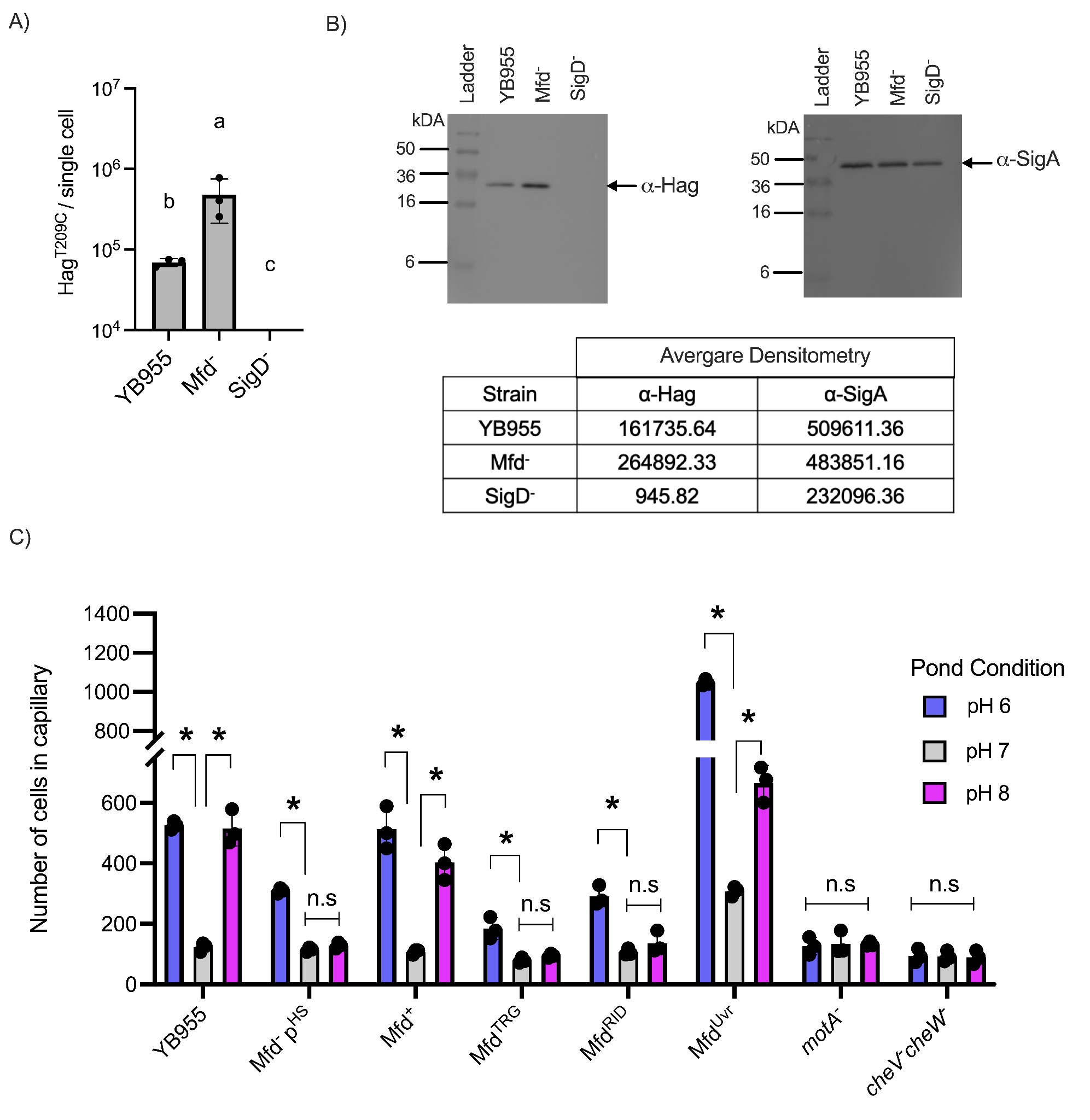
 **Supplemental Figure 5. Effects of Mfd on Hag protein levels and pH taxis in *B. subtilis.*** A) Quantification of Hag^T209C^ fluorescence levels (s*ee methods*) per single cell, normalized to cell-length.B) Cytoplasmic protein levels of Hag and SigA in stationary-phase YB955, Mfd, and SigD-deficient strains via western blotting. The average densitometry analysis of the corresponding band from each condition is reported. C) Results from the capillary-based pH taxis assay. Cells were inoculated into liquid “ponds” adjusted to acidic (pH 6), alkaline (pH 8), or optimal (pH 7) conditions. A capillary tube containing a neutral buffer (pH 7) was inserted into each pond. Cells in the capillary tube were plated to determine colony-forming units (CFUs) as a readout of chemotactic response. The strains tested include the parent YB955, Mfd-deficient, complemented (Mfd⁺), as well as mutant alleles of *mfd,* G977R (Mfd^TRG^), L522R (Mfd^RID^), and R177A (Mfd^Uvr^). Controls include a *motA*-deficient strain and a double *cheV cheW* mutant. Dots represent biological replicates; error bars represent standard deviation. Lowercase letters denot significance between means, and (*) correspond to P < 0.05, and "n.s." denotes non-significant by one-way ANOVA with LSD test.

**References**

1. Martin, H.A., et al., *Mfd Affects Global Transcription and the Physiology of Stressed Bacillus subtilis Cells*. Front Microbiol, 2021. 12: p. 625705.

2. Deaconescu, A.M., et al., *Structural basis for bacterial transcription-coupled DNA repair.* Cell, 2006. 124(3): p. 507-20.

3. Le, T.T., et al., *Mfd Dynamically Regulates Transcription via a Release and Catch-Up Mechanism. Cell,* 2018. 172(1-2): p. 344-357 e15.
